# 2302. Transmissibility of SARS-CoV-2 by symptom: data from a case-ascertained household transmission study

**DOI:** 10.1093/ofid/ofad500.1924

**Published:** 2023-11-27

**Authors:** Alexandra Mellis, Sarah E Smith-Jeffcoat, Joshua Petrie, H Keipp Talbot, Kerry Grace Morrissey, Melissa Stockwell, Yvonne A Maldonado, Natalie M Bowman, Karen Lutrick, Suchitra Rao, Phillip P Salvatore, Jessica E Biddle, Vanessa Olivo, Steph Battan-Wraith, Lori S Merrill, Son H McLaren, Ellen Sano, Anny Diaz, Clea Sarnquist, Prasanthi Govindaranjan, Sara H Goodman, Katherine Ellingson, Karla I Ledezma, Kathleen Pryor, Jessica T Lin, Ayla Bullock, Amy Yang, Edward Belongia, Huong McLean, Edwin J Asturias, Hector Izurieta, Kimberly W Hart, Jonathan Schmitz, Yuwei Zhu, Melissa A Rolfes, Carlos G Grijalva

**Affiliations:** Centers for Disease Control and Prevention, Atlanta, GA; Centers for Disease Control and Prevention, Atlanta, GA; Marshfield Clinic Research Institute, Marshfield, Wisconsin; Vanderbilt University Medical Center, Nashville, Tennessee; Westat, Rockville, Maryland; Columbia University Irving Medical Center, New York City, New York; Stanford University, Stanford, California; University of North Carolina, Chapel Hill, North Carolina; University of Arizona College of Medicine, Tucson, Arizona; University of Colorado School of Medicine, Aurora, Colorado; Centers for Disease Control and Prevention, Atlanta, GA; Centers for Disease Control and Prevention, Atlanta, GA; Westat, Rockville, Maryland; Westat, Rockville, Maryland; Westat, Rockville, Maryland; Columbia University Irving Medical Center, New York City, New York; Columbia University Irving Medical Center, New York City, New York; Columbia University Irving Medical Center, New York City, New York; School of Medicine, Stanford University, Palo Alto, California; Stanford University School of Medicine, Stanford, California; Stanford University School of Medicine, Stanford, California; University of Arizona, Tucson, Arizona; University of Arizona College of Medicine, Tucson, Arizona; University of Arizona College of Medicine, Tucson, Arizona; University of North Carolina at Chapel Hill, Chapel Hill, North Carolina; University of North Carolina, Chapel Hill, North Carolina; University of North Carolina, Chapel Hill, North Carolina; Marshfield Clinic Research Institute, Marshfield, Wisconsin; Marshfield Clinic Research Institute, Marshfield, Wisconsin; University of Colorado School of Medicine, Aurora, Colorado; Food and Drug Administration, Bethesda, Maryland; Vanderbilt University Medical Center, Nashville, Tennessee; Vanderbilt University Medical Center, Nashville, Tennessee; Vanderbilt University, Nashville, Tennessee; Centers for Disease Control and Prevention, Atlanta, GA; Vanderbilt University Medical Center, Nashville, Tennessee

## Abstract

**Background:**

Understanding the transmissibility of respiratory viruses by symptoms is important for public health.

**Methods:**

Persons who tested positive for SARS-CoV-2 and their household contacts (HHC) were recruited from 7 US sentinel sites or by remote invitation nationwide during Sep. 2021—Mar. 2023. The household primary case was the person with the earliest symptom onset or positive test. Starting ≤6 days after primary case onset, primary cases and HHC completed symptom logs (daily, retrospective since onset and for 10 days post-enrollment) and collected nasal or saliva specimens (daily for 10 days) that were tested by RT-PCR. Infected individuals were counted as having developed fever, lower respiratory symptoms (LRS: wheezing, chest tightness/pain, shortness of breath, cough), other symptoms (fatigue, aches, abdominal pain, diarrhea, vomiting, change of taste/smell, headache, sore throat, runny nose, nasal congestion), or as being asymptomatic based on all logs. Risk of secondary infection (any PCR positivity) among eligible, tested HHC (Methods 1) by symptoms of primary cases was estimated using Poisson regression with generalized estimating equations. We estimated days from onset to last PCR positive in a survival model.

Methods upload 1. Enrolled and analytically included household members in case-ascertained studies of household transmission of SARS-CoV-2, United States, Sept 2021 - Mar 2023.

**Figure:**
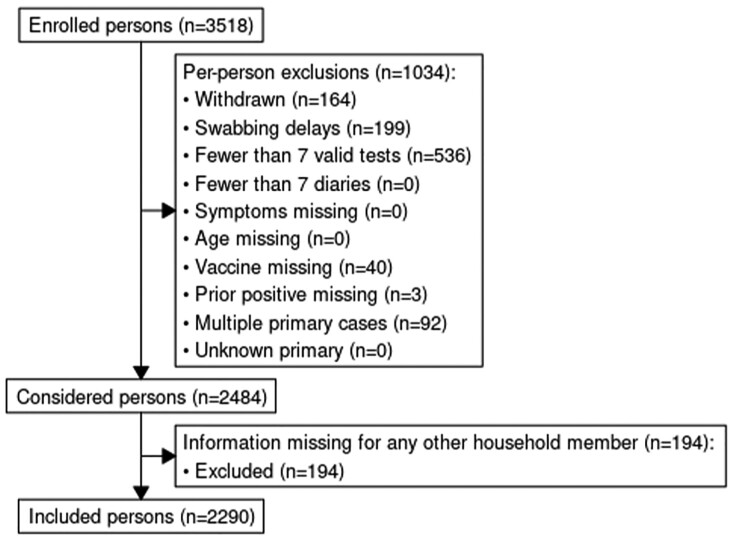

**Results:**

This analysis included 842 households (839 primary cases, 836 infected HHC, and 615 uninfected HHC, median household size of 2). Most primary cases (99%) and infected HHC (81%) were symptomatic (Results 1). Primary cases had higher frequencies of fever or LRS than infected HHC (Results 2). HHC exposed to primary cases who developed fever or LRS were more likely to become infected than HHC exposed to primary cases who did not have fever or LRS (Results 3). Post-hoc comparisons by individual symptoms supported this for fever and all LRS but chest pain (fever: IRR 1.31 95% CI: 1.13-1.52; cough: IRR 1.54 95% CI 1.21 – 1.95; wheezing: IRR 1.20 95% CI 1.08 – 1.35; shortness of breath IRR 1.15, 95% CI 1.04 – 1.27). Primary cases with fever or LRS were PCR positive for a median of 14 days (95% CI: 14 – 15) post-onset, compared to 10 days (95% CI: 9 – 11) for cases who did not have fever or LRS.

Results upload 1. Characteristics of included household members in case-ascertained studies of household transmission of SARS-CoV-2, United States, Sep 2021 - Mar 2023.

**Figure:**
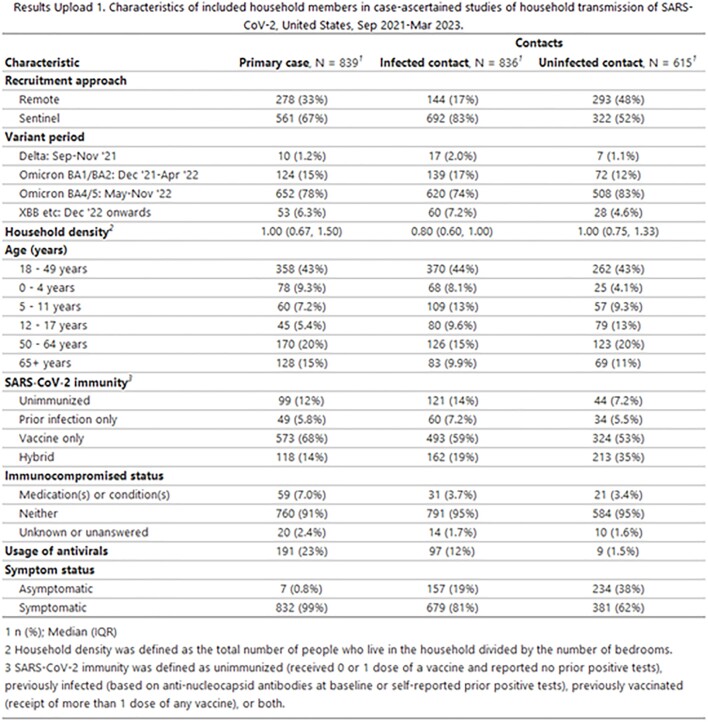

Results upload 2. Proportion of primary cases and infected household contacts who experienced individual symptoms.

**Figure:**
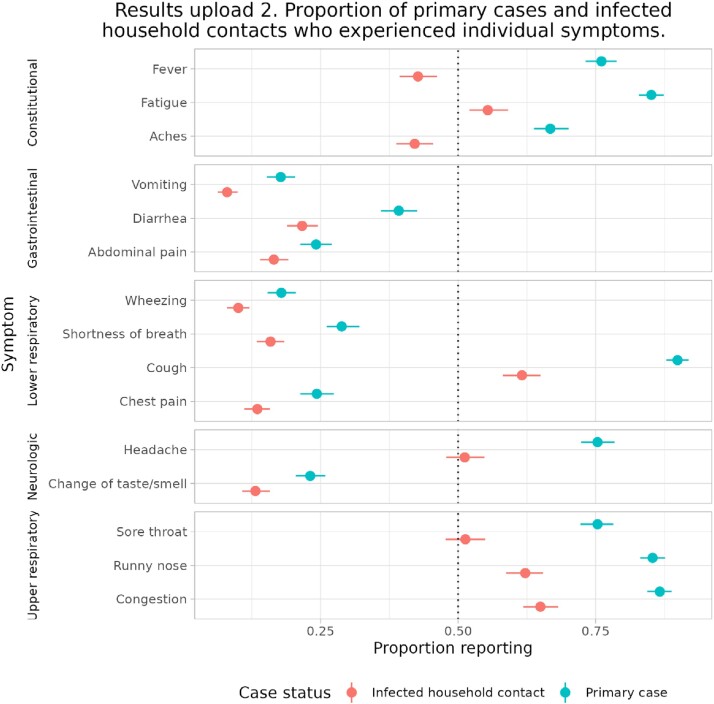

Results upload 3. Unadjusted and adjusted risk of household contacts becoming infected with SARS-CoV-2, by symptoms in the primary case.

**Figure:**
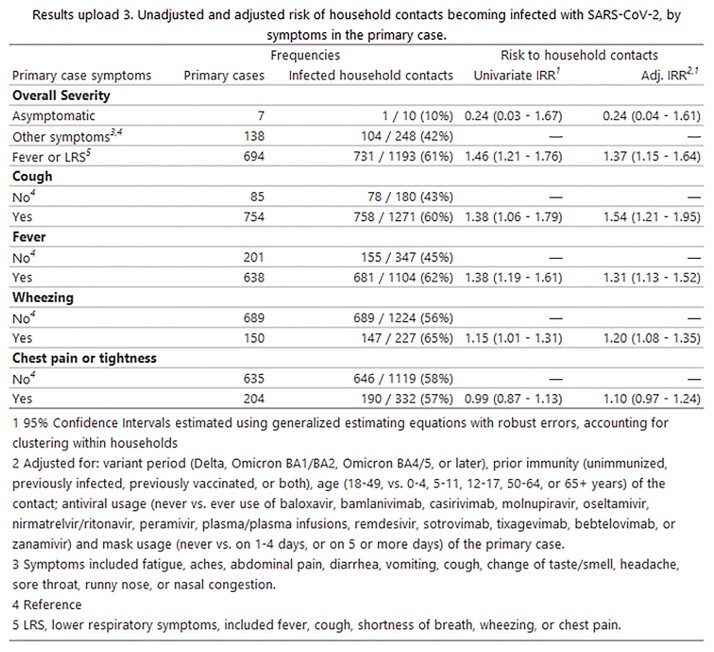

**Conclusion:**

Contacts of primary cases with fever or lower respiratory symptoms may have been more likely to become infected than contacts of primary cases without, suggesting higher transmissibility.

**Disclosures:**

**Joshua Petrie, PhD**, CSL Seqirus: Grant/Research Support **Yvonne A. Maldonado, MD**, Pfizer: Grant/Research Support|Pfizer: Site Investigator, DSMB member **Suchitra Rao, MBBS, MSCS**, Sequiris: Advisor/Consultant **Edward Belongia, MD**, Seqirus: Grant/Research Support **Huong McLean, PhD, MPH**, Seqirus: Grant/Research Support **Edwin J. Asturias, MD**, Hillevax: Advisor/Consultant|Moderna: Advisor/Consultant|Pfizer: Grant/Research Support **Carlos G. Grijalva, MD, MPH**, AHRQ: Grant/Research Support|CDC: Grant/Research Support|FDA: Grant/Research Support|Merck: Advisor/Consultant|NIH: Grant/Research Support|Syneos Health: Grant/Research Support

